# Distinct Mechanisms for Processing Autophagy Protein LC3‐PE by RavZ and ATG4B

**DOI:** 10.1002/cbic.202000359

**Published:** 2020-08-25

**Authors:** Aimin Yang, Supansa Pantoom, Yao‐Wen Wu

**Affiliations:** ^1^ School of Life Sciences Chongqing University 401331 Chongqing P. R. China; ^2^ Translational Neurodegeneration Section “Albrecht-Kossel” Department of Neurology University Medical Center Rostock 18147 Rostock Germany; ^3^ Department of Chemistry Umeå Centre for Microbial Research Umeå University 90187 Umeå Sweden; ^4^ Chemical Genomics Centre of the Max Planck Society Otto-Hahn-Strasse 15 44227 Dortmund Germany; ^5^ Max Planck Institute of Molecular Physiology Otto-Hahn-Strasse 11 44227 Dortmund Germany

**Keywords:** autophagy, cysteine proteases, expressed protein ligation, *Legionella pneumophila*, peptide synthesis

## Abstract

Autophagy is a conserved catabolic process involved in the elimination of proteins, organelles and pathogens in eukaryotic cells. Lipidated LC3 proteins that are conjugated to phosphatidylethanolamine (PE) play a key role in autophagosome biogenesis. Endogenous ATG4‐mediated deconjugation of LC3‐PE is required for LC3 recycling. However, the *Legionella* effector RavZ irreversibly deconjugates LC3‐PE to inhibit autophagy. It is not clear how ATG4 and RavZ process LC3‐PE with distinct modes. Herein, a series of semisynthetic LC3‐PE proteins containing C‐terminal mutations or insertions were used to investigate the relationship of the C‐terminal structure of LC3‐PE with ATG4/RavZ‐mediated deconjugation. Using a combination of molecular docking and biochemical assays, we found that Gln116, Phe119 and Gly120 of LC3‐PE are required for cleavage by both RavZ and ATG4B, whereas Glu117(LC3) is specific to cleavage by RavZ. The molecular ruler mechanism exists in the active site of ATG4B, but not in RavZ. Met63 and Gln64 at the active site of RavZ are involved in accommodating LC3 C‐terminal motif. Our findings show that the distinct binding modes of the LC3 C‐terminal motif (116–120) with ATG4 and RavZ might determine the specificity of cleavage site.

## Graphical abstract

**Cut autophagy**: Semisynthetic lipidated autophagy protein LC3‐PE enables biochemical and structural analysis of the protein's structure‐function relationship. The distinct binding modes of the LC3 C‐terminal motif might determine the specificity of cleavage by endogenous ATG4 and the *Legionella* effector RavZ.

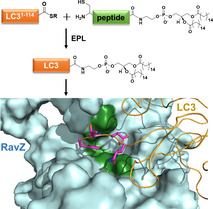

## Introduction

Autophagy is an evolutionarily conserved “self‐eating” process to eliminate damaged organelles and proteins in eukaryotes. It plays an essential role in cellular homeostasis in response to various environmental and cellular stresses. During autophagy the cytosolic components are sequestered within the double‐membrane autophagosome, which fuses with a lysosome for degradation. Autophagy has been associated with diverse human diseases, including cancer, neurodegeneration and pathogen infection.^[1]^

Microtubule‐associated protein light chain 3 (LC3) and GABARAP family proteins are the mammalian orthologs of yeast Atg8, which are required for the biogenesis of autophagosomes and need to be C‐terminally conjugated to phosphatidylethanolamine (PE) for membrane association and function. Newly synthesized pro‐LC3 is cleaved by a cysteine protease, ATG4, to expose a C‐terminal glycine. The processed LC3 serves as a substrate in a ubiquitin‐like conjugation reaction catalyzed by E1‐like ATG7, E2‐like ATG3 and the E3‐like ATG12‐ATG5‐ATG16L complex, which mediate the formation of an amide bond between the carboxyl group of C‐terminal glycine and the amino group of PE in the membrane.^[2]^ The ATG16L complex is generated by another ubiquitin‐like conjugation system. ATG12 is conjugated to the lysine side chain of ATG5 in sequential reactions catalyzed by ATG7 and ATG10.^[3]^ ATG4 deconjugates ATG8/LC3‐PE from the outer surface of autophagosomes to promote autophagosome maturation.^[4]^

Autophagy serves as a defense mechanism to combat infection with pathogenic microbes.^[5]^ On the other hand, bacteria have also evolved diverse mechanisms to battle autophagy for survival.^[6]^ The intracellular bacterium *Legionella pneumophila* disrupts autophagy and escapes from host autophagic degradation. The *Legionella* effector RavZ is injected into the cell and functions as a cysteine protease that irreversibly deconjugates LC3‐PE to inhibit autophagosome formation.^[7]^ Unlike endogenous ATG4 that cleaves the amide bond between terminal glycine and PE, RavZ cleaves the amide bond before glycine. As a consequence, the RavZ‐cleaved LC3 proteins cannot be relipidated, leading to inhibition of autophagosome formation. Our previous work revealed a “lift and cut” mechanism that RavZ extracts LC3‐PE from the membrane before deconjugation.^[8]^ However, it remains unclear how ATG4 and RavZ process LC3‐PE in distinct manners.

In this work, we prepared semisynthetic LC3‐PE proteins with mutations and insertions in the C‐terminal motif (116–120) by expressed protein ligation (EPL). These semisynthetic LC3‐PE proteins were used to investigate the relationship of the C‐terminal structure of LC3‐PE with the ATG4/RavZ‐mediated deconjugation. Our results revealed that the C‐terminal motif of LC3 is critical for RavZ recognition and activity. We identified the amino acid residues at the active site of RavZ involving in the binding of LC3 C‐terminal motif. The distinct binding modes of LC3 C‐terminal motif with ATG4 and RavZ might determine different cleavage sites.

## Results

### C‐terminal residues of LC3‐PE are required for RavZ‐ or ATG4B‐mediated cleavage

**Figure 1:**
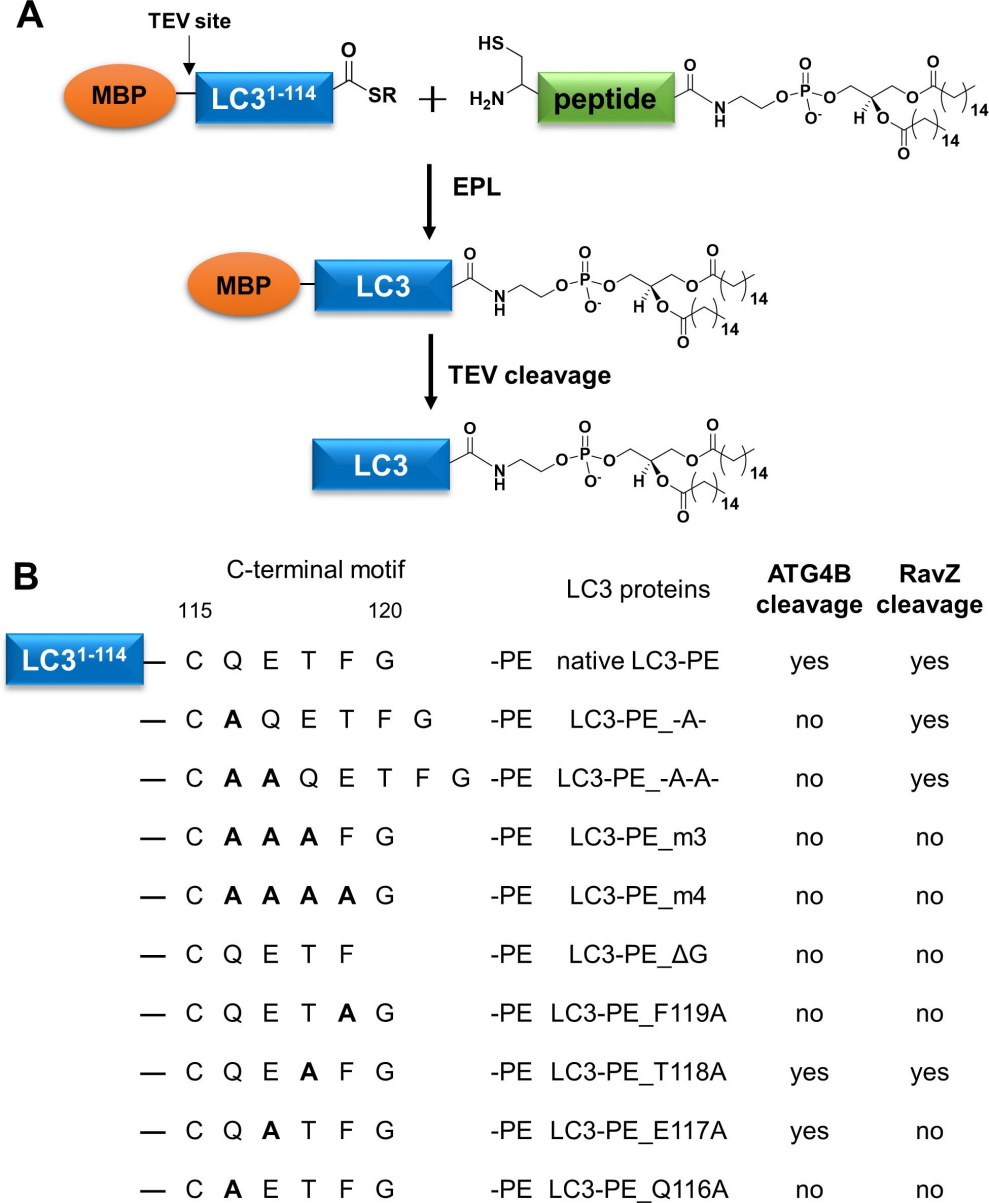
Semisynthetic LC3‐PE proteins and their activity toward RavZ and ATG4B. A) Semisynthesis of LC3‐PE proteins containing different C‐terminal mutations by EPL. B) A summary of ATG4B and RavZ cleavage for all LC3‐PE proteins.

**Figure 2:**
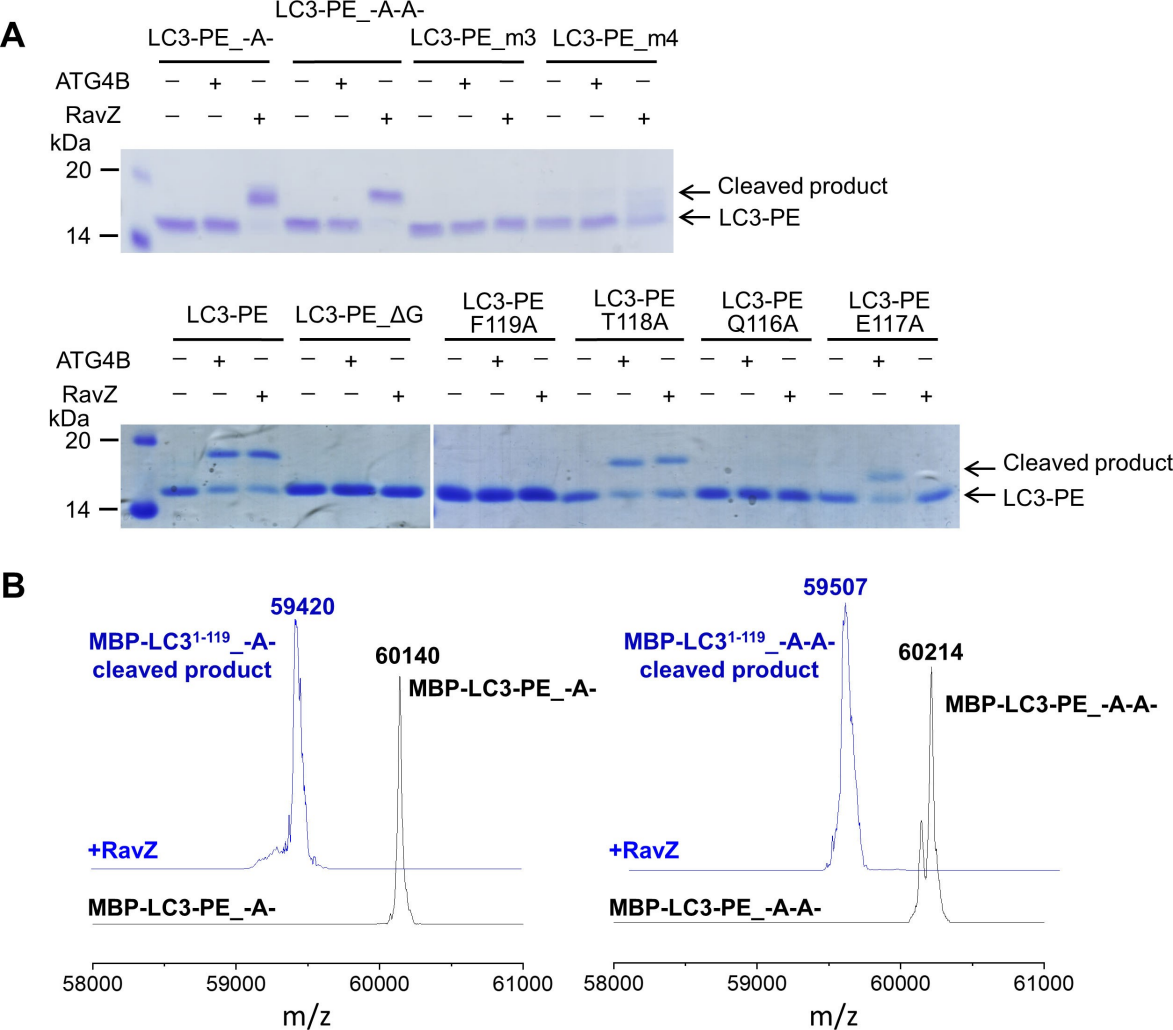
RavZ and ATG4B reaction with LC3‐PE containing different C‐terminal mutations. A) SDS‐PAGE assay for the cleavage of LC3‐PE proteins. The LC3‐PE proteins were treated with RavZ or ATG4B and then analyzed by SDS‐PAGE (top). B) ESI‐MS spectra of MBP‐LC3‐PE_A and MBP‐LC3‐PE_A−A before and after RavZ‐cleavage. MBP‐LC3‐PE_A, *M*_w_ calcd.: 60152, found: 60140; MBP‐LC3^1−119^_A, *M*_w_ calcd.: 59421, found: 59420. MBP‐LC3‐PE_A−A, *M*_w_ calcd.: 60223, found: 60214; MBP‐LC3^1−119^_A−A, *M*_w_ calcd.: 59492, found: 59507.

The ATG4B‐LC3 complex revealed that the C‐terminal motif (116–120) of unlipidated LC3 is crucial for recognition by ATG4B.^[9]^ To examine the structure–function relationship between the C‐terminal sequence of LC3‐PE and RavZ/ATG4B activity, we prepared semisynthetic LC3‐PE proteins with C‐terminus mutations or additional amino acids using a combination of lipidated peptide synthesis and expressed protein ligation (EPL; Figure 1A).^[10]^ The semisynthetic LC3‐PE proteins were treated with RavZ or ATG4B. The cleavage was monitored by SDS‐PAGE and ESI‐MS. LC3‐PE proteins with additional Ala or double Ala insertion between positions 115 and 116 can be processed by RavZ but not by ATG4B (Figures 1B and 2A), suggesting that the flexibility of the active site of ATG4B and RavZ for accommodating LC3 C‐terminal sequence are quite different. Interestingly, the insertion does not lead to alternation of the cleavage site (between position 119 and 120) on LC3‐PE (Figure 2B). This result indicates that the specific processing site is determined by the association of LC3 C‐terminal motif (116–120) with the active site of RavZ.

Next, we mutated the C‐terminal residues (QET or QETF) to Ala or truncated Gly120. Neither RavZ nor ATG4B could process these LC3‐PE proteins (Figures 1B and 2A). Alanine scanning shows that Gln116, Phe119 and Gly120 of LC3‐PE are required, whereas Thr118 is not essential for cleavage by RavZ and ATG4B. However, Glu117 is crucial for the cleavage by RavZ but not for ATG4B (Figures 1B and 2A). The results are consistent with the previous report on the processing of unlipidated LC3 by ATG4B, suggesting that the C‐terminal motif of LC3‐PE associates with ATG4B in the same way as unlipidated LC3.^[9]^ These findings suggest that the LC3 C‐terminal motif binds to the active site of RavZ in a distinct manner from the ATG4B‐LC3 interaction.

### Molecular docking shows the interaction of RavZ with LC3 C‐terminal motif

**Figure 3:**
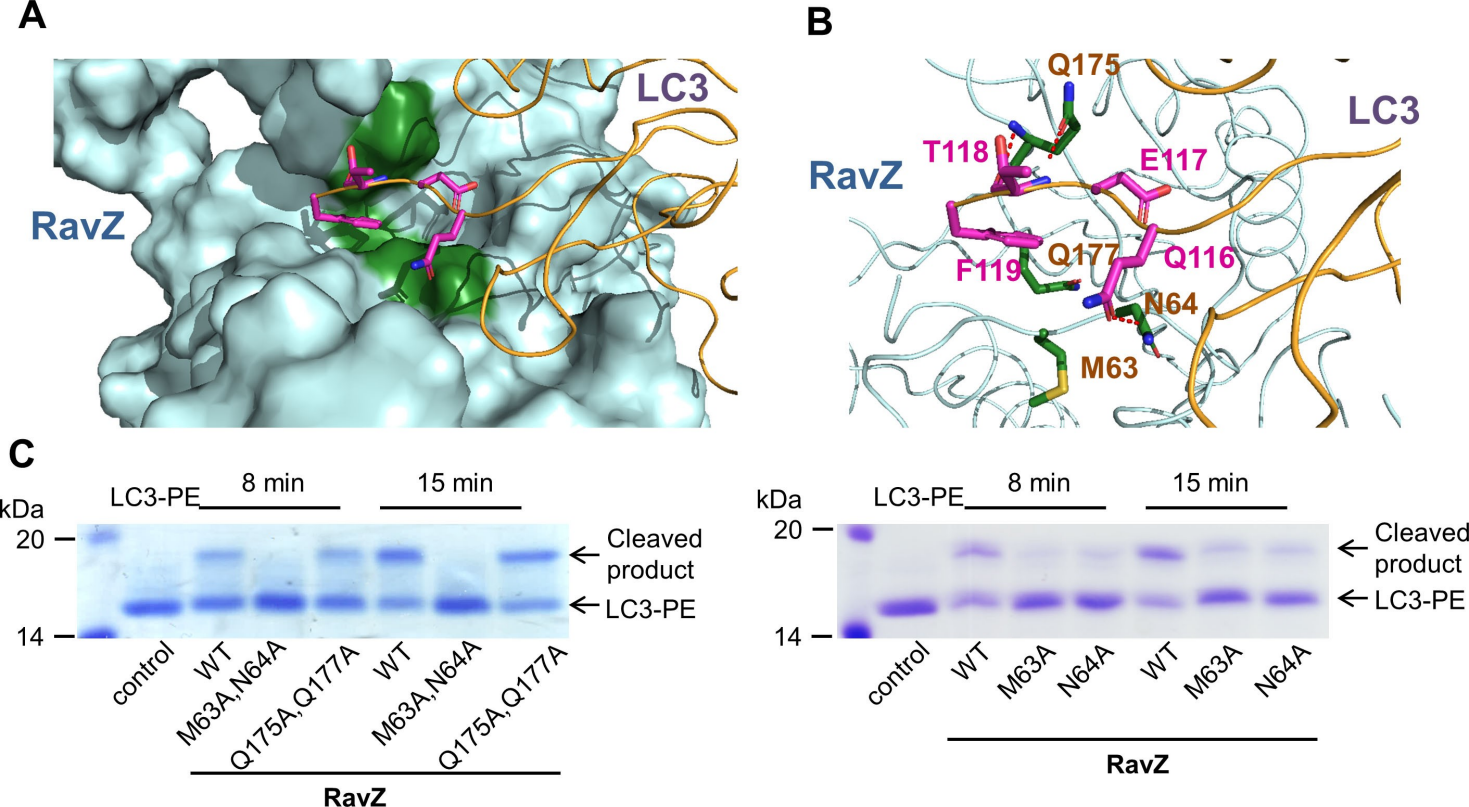
Identification of amino acid residues of RavZ involved in the binding of the LC3 C‐terminal motif. A) Surface representation of the molecular docking of RavZ‐LC3. B) Interaction of the C‐terminal motif of LC3 with the active site of RavZ. The binding residues on LC3 and the active site of RavZ are shown as magenta and green sticks, respectively. The hydrogen bonds are shown as red dashed lines. C) SDS‐PAGE assay for cleavage of LC3‐PE proteins by RavZ WT or mutants.

To further understand the atomic interaction of the C‐terminal motif of LC3 with RavZ, we employed the protein–protein molecular docking to simulate the interaction at the RavZ active site, as the structural data of the RavZ‐LC3‐PE complex is still unknown. The docking revealed three main interacting sites of LC3 C‐terminal motif with the active site of RavZ. The first is hydrophobic interaction of Phe119 of LC3 with Met63 and Gln177 of RavZ. The second is the hydrogen bonding between the amide group in the main chain of Thr118 on LC3 and Gln175 of RavZ. The third is the hydrogen bonding between the side chains of Gln116 on LC3 and Asn64 of RavZ (Figure 3A and B). The docking results suggest that the residues Gln116 and Phe119 of LC3 and the residues Met63, Asn64 and Gln175 of RavZ might possibly be involved in the RavZ‐LC3 interaction, whereas the Glu117 of LC3 did not show significant interaction with the active site of RavZ in the simulation. However, *in vitro* cleavage assay demonstrated that the Glu117 is the specific residue for the cleavage by RavZ but not ATG4B, suggesting that this residue is involved in the interaction with RavZ.

### Validation of the interaction between RavZ and the LC3 C‐terminal motif

To identify the key residues at the active site for RavZ activity, the potential LC3‐interacting residues on RavZ, Met63, Asn64, Gln175 and Gln177 were mutated to Ala. Firstly, the double mutants were used for RavZ cleavage assay. RavZ (M63A, N64A) did not show activity for LC3‐PE protein, whereas RavZ (Q175A, Q177A) could process LC3‐PE as RavZ wild type (Figure 3C). Then, LC3‐PE protein was treated with RavZ proteins with single mutations. Moreover, the single mutants RavZ M63A and N64A showed reduced activity to LC3‐PE (Figure 3C). Therefore, our biochemical results suggested that the molecular docking partially reflects the interaction of RavZ with the C‐terminal motif of LC3‐PE. Met63 and Asn64 of RavZ are probably involved in the binding with Phe119 and Gln116 of LC3, respectively.

## Discussion

RavZ cleaves the amide bond between Phe119 and Gly120 of LC3‐PE. In contrast, ATG4B hydrolyzes the amide bond between Gly120 and PE. This is an important feature of the *Legionella* effector RavZ, leading to irreversible inhibition of LC3 lipidation. To understand why RavZ and ATG4B act at different cleavage sites, we made LC3‐PE proteins with mutations at the C‐terminal motif by a semisynthetic approach. Such a chemical approach renders it possible to prepare LC3‐PE mutants, which are not amenable by the enzymatic approach using the recombinant ATG12‐ATG5‐ATG16L system. Therefore, we were able to investigate the structure‐activity relationship.

**Figure 4:**
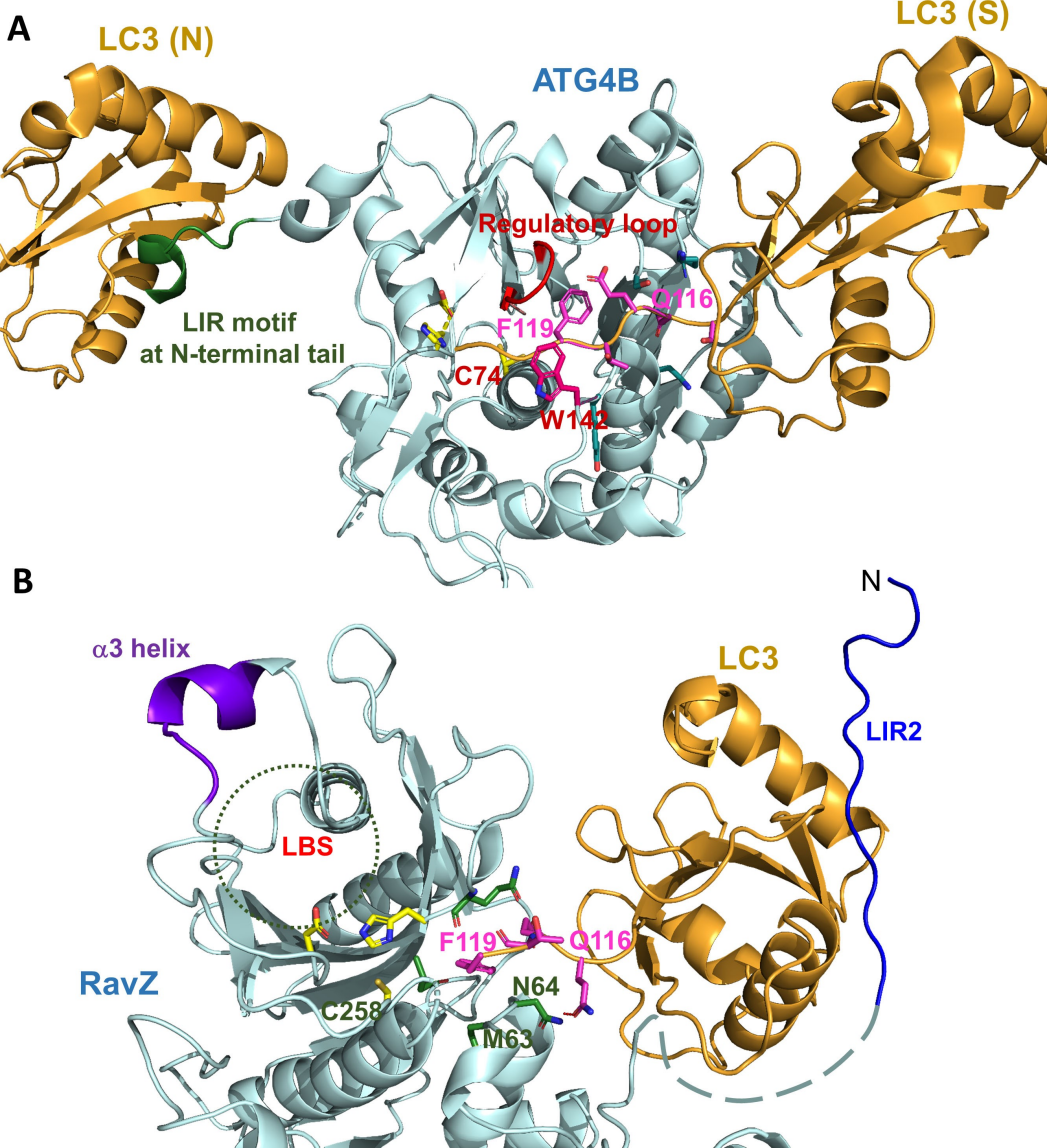
Binding modes of LC3 with ATG4B and RavZ. A) Interaction of the N‐terminal tail of ATG4B with the non‐substrate LC3B (LC3B (N)) opens the active site of ATG4B. Therefore, Phe119 of the substrate LC3B (LC3B (S)) could recognize the active site of ATG4B by interaction with the regulatory loop and replace Trp142 of ATG4B. Gln116, Phe119 and Gly120 in the C‐terminal motif of LC3 are involved in binding to ATG4B. The catalytic triad residues of ATG4B are shown as yellow sticks. The regulation loop and the Trp142 side chain of ATG4B are shown in red. The side chains of LC3 C‐terminal motif are shown as magenta sticks. B) Interaction of RavZ with LC3‐PE consists of LIR2 with the LIR binding site of LC3, LBS with the conjugated PE moiety, and the C‐terminal motif of LC3 with the active site of RavZ. The latter involves Gln116, Glu117, Phe119 and Gly120 of LC3 and Met63 and Gln64 of RavZ, which are shown as magenta and green sticks, respectively. The catalytic triad residues of RavZ are shown as yellow sticks.

The insertion of additional residues at the C‐terminal motif of LC3 abrogates the cleavage of LC3‐PE by ATG4B. This finding suggests that ATG4B‐mediated cleavage is governed by a molecular ruler mechanism, which monitors the length and the identity of the C‐terminal motif 5 residues away from the cleavage site. Moreover, the specificity of ATG4B is endowed by the regulatory loop and Trp142 of ATG4B.[^9^, ^11^] LC3 binding results in the replacement of the side chain of Trp142 by Phe119 of LC3 that pushes the regulatory loop to the open conformation. This leads to the formation of a narrow groove that can only accommodate a glycine residue at the entrance to the active site (Figure 4A). Therefore, Phe119 and Gly120 of LC3 are essential for the processing by ATG4B. These residues are also important for RavZ cleavage. However, such a regulatory loop and analogous Trp residue are not observed in RavZ. The molecular ruler mechanism does not exist in RavZ, either. Our results suggest that Phe119 of LC3 may be involved in hydrophobic interaction with Met63 of RavZ. Both residues are required for RavZ‐mediated cleavage of LC3‐PE. The putative hydrogen bonding interaction between the side chain of Gln116 on LC3 and Asn64 of RavZ appears to be important for RavZ‐mediated cleavage. Whereas in the ATG4B‐LC3 complex, Gln116 only interacts with the main chain of ATG4B (Figure 4A). Glu117 of LC3 is crucial for the cleavage by RavZ but not for ATG4B. The biochemical analysis showed that Met63 and Gln64 at the active site of RavZ play an important role for accommodating the C‐terminal motif of LC3 (Figure 3). Our previous research showed that the second N‐terminal LIR (LIR2) motif and the lipid binding site (LBS) of RavZ bind to the LIR‐binding site of LC3 and the conjugated PE moiety, respectively (Figure 4B). These interactions are indispensable for RavZ‐mediated cleavage of LC3‐PE.^[8a]^ They may facilitate the association of the C‐terminal motif of LC3 with the active site of RavZ. Consequently, more flexibility in the sequence upstream of the C‐terminal motif (116–120) of LC3 is made possible (Figure 1B). The specificity of the cleavage site is probably defined by the specific binding mode between LC3‐PE and RavZ. Presumably, the distinct binding modes of LC3 C‐terminal motif with ATG4B and RavZ contribute to the specificity of the cleavage site (Figure 4).

In conclusion, using semisynthetic LC3‐PE proteins we revealed that LC3 C‐terminal motif is critical for the recognition by RavZ (Figure 1). Based on the results shown here together with previous findings,^[8]^ the working model for RavZ is reinforced. RavZ initially binds LC3 on phagophore membrane through the LIR motif. Then, PE moiety of LC3‐PE is recognized by α3 helix of RavZ and its lipid group is docked into the LBS of RavZ. LC3 C‐terminal motif binds to the active site of RavZ. The LIR binding, the lipid binding and the interaction of LC3 C‐terminal motif facilitate the correct orientation and specific binding of the active site of RavZ to the C‐terminal motif of LC3‐PE. Finally, enzymatic activity of RavZ causes deconjugation of LC3‐PE at a specific site between Phe119 and Gly120. Our results provide new insights into the distinct molecular mechanisms for the processing of LC3‐PE by the *Legionella* effector RavZ and by the endogenous ATG4B.

## Experimental Section

**Plasmids**: Using site‐directed mutagenesis with one primer, RavZ mutations were obtained by the pMAL vector carrying RavZ. Briefly, RavZ mutations were amplified by PCR using a pMAL‐RavZ plasmid as a template. The following primers were used: 5′‐ACCAGCTGGGAAGTGAATAAAGGGGCGGCGAGTTCTCGTTTGCATAAACTA‐3′ (f) for RavZ (M63A, N64A), 5′‐ACCAGCTGGGAAGTGAATAAAGGGGCGAACAGTTCTCGTTTGCATAAACTA‐3′ (f) for RavZ (M63A), 5′‐ACCAGCTGGGAAGTGAATAAAGGGATGGCGAGTTCTCGTTTGCATAAACTA‐3′ (f) for RavZ (N64A), 5′‐GACATTACCAAAGGGGTTGCGCACGCGGTATTGTTAACTATTAGCTACGAT‐3′ (f) for RavZ (Q175A, Q177A). The PCR product was treated with DpnI and further transformed into the XL1 blue competent cells. After incubation overnight in the Agar plate, the several clones were cultured and miniprepared. The positive clones were validated by DNA sequence.

**Synthesis of LC3‐PE proteins**: The lipidated proteins were achieved by performing express protein ligation (EPL) of recombinant LC3‐thioester proteins with synthetic lipidated peptides. The synthesis has been described in details elsewhere.^[10]^

**Protein expression and purification**: Plasmid containing RavZ (or mutant) or ATG4B was transformed into *Escherichia coli* BL21(DE3)‐LIR cells. Protein expression was induced with 0.2–0.4 mM IPTG and carried out at 20 °C overnight. Purification was performed with the Äkta prime plus chromatography purification system (GE Healthcare Life Sciences). The cells were harvested and resuspended in breaking buffer containing 1× protease cocktail (Roche Life Science) or 1 mM PMSF. Cells were then lysed by a Microfluidizer (Microfluidics). All of the proteins used in this study contain affinity tags (MBP or GST) with an extra N‐terminal His_6_ tag. The proteins were initially purified by Ni‐NTA affinity purification using HisTrap HP column (GE Healthcare Life Sciences) eluted with a gradient of 0–100 % of 500 mM imidazole. To release the protein from the affinity tags, the fusion proteins carrying precision protease or TEV protease cleavage site were cleaved by the corresponding proteases overnight at 4 °C. The cleaved tag and protease were removed by the HisTrap HP column, proteins were further purified with size exclusion chromatography using the HiLoad 26/60 Superdex 200 column.

**In vitro proteolytic cleavage**: The MBP‐LC3‐PE protein or mutants were treated with tobacco etch virus (TEV) protease (0.06 mg TEV protease to 1 mg MBP‐LC3‐PE) at 4 °C overnight to remove the MBP tag. The resulting LC3‐PE proteins were solubilized in the buffer (20 mM HEPES, pH 7.2, 30 mM NaCl, 1 mM DTT, 0.1 % Triton X‐100). LC3‐PE proteins were treated with RavZ or ATG4B in the buffer (20 mM HEPES pH 7.2, 30 mM NaCl, 1 mM DTT, 0.1 % Triton X‐100) and then analyzed by SDS‐PAGE. LC3‐PE proteins (7 μM) containing additional amino acids, triple (m3) or quadruple mutations (m4) were treated with RavZ (350 nM) or ATG4B (350 nM) at 37 °C for 2 h. LC3‐PE proteins containing a single mutation (7 μM) were treated with RavZ (350 nM) at 37 °C for 15 min or ATG4B (350 nM) at 37 °C for 40 min. For the validation of the interactions of RavZ and LC3 C‐terminal motif, LC3‐PE protein (7 μM) was treated with RavZ WT or mutant (350 nM) at 37 °C for 8 or 15 min.

**MS analysis of RavZ cleavage product**: The MBP‐LC3‐PE protein (7 μM) containing different mutations were treated with RavZ (350 nM) at 37 °C for 2 h and then tested by LC‐MS. LC‐MS analysis of RavZ cleavage product was performed on an Agilent 1100 series chromatography system equipped with an LCQ electrospray mass spectrometer (Finnigan, San Jose, USA) using Jupiter C4 columns (5 μm, 15×0.46 cm, 300 Å pore‐size) from Phenomenex (Aschaffenburg, Germany). Data evaluation was carried out using the Xcalibur software package and MagTran software programs were used for deconvolution of ESI mass spectra of the proteins.

**Protein‐protein molecular docking**: Protein‐protein docking of RavZ‐LC3 was performed using The ZDOCK server.^[12]^ RavZ (PDB ID: 5MS7) and LC3B from ATG4B‐LC3B complexed (PDB ID: 2Z0D) were used as an input for the docking. The docking area is defined from the residues C258, D197 and H176 in the active site of RavZ and residues N116, E117, T118, F119, G120 from the C‐terminal of LC3B. The docking structure of RavZ‐LC3B complexed was further refined using the local search refinement of the Rosetta Dock server.^[13]^

## Conflict of interest

The authors declare no conflict of interest.
